# Remarks on Vivisection, and on Certain Allegations as to Its Utility in the Study and Application of Physiology

**Published:** 1847-10-01

**Authors:** 


Remarks on Vivisection, and on certain Allegations as to its Utility and Necessity in the Study and Application of Physiology. By *George Macilwain*, F.R.C.S., &c. &c. 8vo. pp. 27. London, 1847.

This is an excellent pamphlet, well argued and very gracefully written. It contains much that deserves the serious attention of the truly philosophic, as well as of the purely benevolent, physician. The author, not satisfied with protesting against the cruelty, exposes the worthlessness in a mere scientific point of view, of *many* (we do not say *all*) of the experiments on living animals, which have at different times been adduced in favour of the practice he condemns. He comments more particularly on certain experiments of Hunter, Orfila, Sir A. Cooper, and Sir C. Bell.

It has been often alleged that the great improvement, which Hunter introduced into the treatment of aneurism by operation, was the result of this mode of investigation. That such was not the case, but that the happy thought of tying the artery at some distance from the seat of disease sprung solely and entirely from the observation of pathological phenomena—we allude more particularly to his knowledge of the fact that " the sac often by it increase presses on the sound part of the artery and becomes the cause of its obliteration," whereas the diseased portion of the vessel, when encircled with a ligature, will seldom unite—and from the simple deductions of legitimate reasoning thereon, is clearly made out by referring to the history of the very case which led Hunter to its adoption. It is given at page 10 of the " Remarks."

Mr. Macilwain's observations on Orfila's experiments, which have certainly been among the most barbarous of the present age, are so good that we could wish to have been able to give them entire. We shall select the most striking portions.

" The nature of the experiments, and M. Orfila's narrative, alike demonstrate the tortures by which they were accompanied. He tells us of animals dying after hours of pain, in the midst of the most horrible sufferings, 'au milieu des souffrances les plus horribles, douleurs les plus atroces,' 'poussant des cris les plus plaintives'—'in most dreadful pain,' 'uttering the most plaintive cries,' and similar unequivocal expressions of sustained agony. It would be some little satisfaction could we assert that these expefiments had contributed to the advance of science, but, as will be immediately shewn, this is far from having been the case.

" The experiments varied; those chiefly referred to, consisted in exposing an animal's œsophagus (or gullet), by dissection, making an opening into it, and injecting thereby poison into the stomach; the gullet was then tied ! and the animal left to itself. In some cases the ligature was removed and more poison, or its supposed antidote, injected; other experiments consisted in making a wound, and sewing up some poison in it, to see what effect would be produced. Although the influence on science exerted by these experiments is the point with which we have to deal, we may venture to remark that there were philosophical objections to them, on the very threshold—insuperable, because loaded with so many elements of fallacy. I shall very briefly advert to them: 1st. To ascertain the effect of a poison introduced coincidently with the infliction of a severe operation, it is obviously necessary that we should be able distinctly to separate the disturbance occasioned by the operation, from that resulting from the poison; but this cannot be done, because the same amount of local injury even in animals, by no means always produces the same consequences.

" 2ndly. Every one knows that many substances in nature excite very different effects in dogs, horses, hares, goats, &c., from those which they produce in man.

The torture too (I mean of the operation), as distinct from that resulting from the poison, is objectionable in a philosophical sense, because it places the nervous system in a factitious state, of which system you are really asking the question. What should we think of estimating the natural action of the stomach on any substance in a patient who was ill or in pain from other causes, or in whom we had just tied the œsophagus ! !"

"The highest claim to attention that *any* experiment *on any* animal can have, as regards the physiology of man or its relation to medical science, is its being resolvable into analogy. But analogy to be anything must be real, freed from all things which can distort the implied parallelism; the moment you institute any process which you cannot imitate in man, the parallelism is destroyed, the analogy is at an end; and lastly, no one thinks of reasoning from analogy when he can reason from fact. This we shall show to be practicable in the present instance, on the testimony of Mr. Orfila himself, whose book contains examples of the effects of almost all sorts of poisons on *Man*, and which, in fact, constitute the chief value of his work on the subject. The truth is that accidents, mistakes, murders, and fatal as well as failing suicides, have furnished a series of facts and experiments in man which it is of course impossible, strictly speaking, to repeat in any other animal. With this fact before us, I would ask any man, whatever his sentiments may be on the general question, whether he would, in any case of poison, dare to institute a treatment founded on observation and experiments in animals, whilst he knew of any which had been deduced from observation on man." P. 13.

Mr. M., while he admits the value of M. Orfila's work upon poisons, very truly remarks that its practical value is almost quite independent of the dreadful experiments which it records.

"Let the book," says he, "be read, *excluding first* all the experiments on living animals, and let the conclusion legitimately deducible from the facts enunciated, be noted; then let the experiments on animals be examined, for the purpose of testing their influence on the conclusions which have been noted, and it will be found that they affect them in no way whatever." P. 14.

Who will gainsay the perfect truth of this valuation? If any one does, he has only to compare it with the rival work of Christison, a name of as high repute and authority as that of Orfila, and he will be constrained to admit that the advancement of toxicological science has been infinitely more indebted to the observation of clinical and necroscopic phenomena, and to the results of chemical research, than to all the horrible disclosures of the experimental torture-house.

Passing over our author's strictures on the very unnecessary and most unscientific experiments of Sir A. Cooper, in which he fractured the neck of the thigh-bone in dogs and other animals, with the view of determining the disputed question whether true osseous union ever takes place after the accident when it occurs in the human subject, we shall close our notice of the "Remarks" by quoting the following passage in reference to the opinions of him, who may fairly be deemed the foremost of modern physiologists. Would that his example and his precepts were more generally followed!

"It is interesting," says Mr. Macilwain, "to observe that we not only have the evidence of Sir Charles Bell, as to the sources whence he adduced his own *discoveries*, which, as it will be seen, were altogether independent of any experiments on living animals; but that he has left us the gratification of knowing what his opinions were *generally* on that mode of investigation. After comparing Chemistry and Anatomy, with a view to impress that the former is emphatically a science dealing largely in experiment, the latter, one which depends chiefly on observation: this distinguished physiologist thus proceeds—'Anatomy is already looked on with prejudice; let not its professors *unnecessarily* incur the censures of the humane; *experiments have never been the means of discovery*, and a survey of what has been attempted of late years will *prove* that the opening of living animals has *done more to perpetuate error,* than to confirm the just views taken from Anatomy and the natural motions.' This is perhaps enough, but again Sir Charles Bell observes, 'In a foreign review of my former papers, the results have been considered as a further proof in favour of experiments; they are, *on the contrary, deductions from Anatomy*—and I have had recourse to experiments, not to form my opinions, *but to impress them on others.* It must *be my apology* that my utmost powers of persuasion were lost whilst I urged my statements on the ground of Anatomy alone. I have made few experiments,' &c. Once more I will quote Sir Charles—'Much has been said (he writes) in favour of experiments made by men unbiassed as to the results. The only instances of this which I can allow are, (he is speaking of nerves, illustrative of his own enquiries,) when surgeons cut the nerves on the face in surgical operations. In such operations as those for Tic Douloreux he is indeed unbiassed, and we have seen the result, that after *fifty years of such experience we remained quite ignorant of the functions of these nerves.*'*

"Surely any one who reads these quotations, whatever may be his own views of Vivisection, will scarcely venture to quote Sir Charles Bell in support of it. The fact is, that to see an animal writhe with torture, and on a particular nerve being divided to exhibit manifestations of loss of motion or sensation, was easy, mere 'eye-work,' which could *per se* prove nothing; but diligently to investigate the courses of nerves in the dead animal, to trace the filaments of which they were composed to their respective relations in the brain and spinal marrow, and then to test the suggestions arising out of these proceedings, by careful observation of phenomena in man, whether arising from disease, accident, or surgical operations, was difficult, tedious, and requiring the exercise of the higher faculties, whilst it constituted the only mode of eliciting the truth; and especially Sir Charles' ultimate object, viz. the application of the whole to the explanation and relief of disease." P. 24.

* "Sir Charles Bell's writings strongly suggest that it was the *fallacy* of Vivisection which created his *distrust* of it, as purely as his humanity had engendered his *dislike* of it. In one of his earlier proceedings, after stating the *apparent* result of the experiment, he says, 'but here there was *confusion* because of sensation, *therefore* the animal was instantly destroyed by a blow on the head; in other words, the suffering was put an end to, because sensation obscured the reasoning on the experiment.'—*Bell on the Nerves.* 8vo. edition, 1st part."

